# A systematic review of studies using pedometers as an intervention for musculoskeletal diseases

**DOI:** 10.1186/1471-2474-15-231

**Published:** 2014-07-10

**Authors:** Suliman Mansi, Stephan Milosavljevic, G David Baxter, Steve Tumilty, Paul Hendrick

**Affiliations:** 1 School of Physiotherapy, University of Otago, Dunedin, New Zealand; 2 School of Physiotherapy, University of Saskatchewan, 1121 College Drive, Saskatoon, SK S7N 0 W3, Canada; 3 Division of Physiotherapy Education, The University of Nottingham, Nottingham NG5, UK

**Keywords:** Motion sensors, Physical activity, Motivation tools, Pedometer, Chronic disease, Step counter

## Abstract

**Background:**

Physical activity (PA) plays an important role in the prevention and management of a number of chronic conditions. Aim: to investigate the evidence for effectiveness of pedometer-driven walking programs to promote physical activity among patients with musculoskeletal disorders (MSDs).

**Method:**

A comprehensive systematic review was performed using 11 electronic databases up to 20 February 2014. Keywords and MeSH terms included “musculoskeletal disorders”, “walking”, and “pedometer”. Randomized controlled trials, published in English, that examined the effects of a pedometer-based walking intervention to increase physical activity levels and improve physical function and pain in patients with musculoskeletal disorders were included.

**Result:**

Of the 1996 articles retrieved, seven studies ranging in date of publication from 1998 to 2013 met the inclusion criteria, allowing data extraction on 484 participants with an age range of 40 to 82 years. Interventions lasted from 4 weeks to 12 months and the results across studies showed significant increases in step count (p < 0.05) following the intervention. Across these studies, there was a mean increase in PA of 1950 steps per day relative to baseline. Four studies reported improved scores for pain and/or physical function at the intervention completion point relative to controls.

**Conclusion:**

This study provides strong evidence for the effectiveness of pedometer walking interventions in increasing PA levels for patients with MSDs. Our findings suggest that a combination of interventions is likely to be the most effective strategy to maximize health benefits in the short term. Further research should include larger sample sizes, and longer intervention durations are required to support the role of pedometer walking interventions as a long term intervention for management of musculoskeletal disorders.

## Background

The worldwide prevalence of musculoskeletal disorders (MSDs) is reflected in increasing costs, [1-3] occupational injury, and long term disability [1,2,4,5]. In particular, musculoskeletal pain constitutes an increasing problem in the ageing population [6] and is an important factor underpinning functional limitations [7-9]. Consequently, musculoskeletal disorders have a large impact on activities at work and home, [7,8] and place a considerable burden on the health care system [4].

Physical activity (PA) plays an important role in the prevention and management of a number of chronic conditions, [10-12] such as cardiovascular disease, [13-15] diabetes mellitus, [16] and obesity, [17] and has been shown to reduce premature mortality and improve quality of life [10] in the general population [10,18]. In recent years a number of studies have demonstrated the benefits of promoting an increase in PA to reduce pain and improve quality of life in the adult population with MSDs, [19-21] to reduce musculoskeletal impairment in the elderly, [20] and reduce pain for those with low back pain (LBP) [22], neck pain, and shoulder pain [23]. Physical activity has also been shown to play a role in protecting against later hip fracture in an adult population [24] and reducing the incidence of osteoporotic vertebral fractures in an elderly population [25]. Results from a systematic review also support the effectiveness of PA to treat and prevent a number of chronic disorders [26]. There are numerous modalities available for the management of MSDs, and considerable debate about the most effective interventions; [9] however, increasing PA as part of the overall management approach is a key feature of a range of studies investigating the management of MSDs [9,27].

Walking is deemed to be one of the most effective forms of PA, with little risk of injury among low-activity populations; [28-30] it has been used successfully as an intervention to reduce the burden of a number of chronic diseases including hypertension, [31] cardiovascular risk, [32] obesity, [33] and osteoarthritis (OA) [34]. Currently, there are a number of studies that support the use of walking-based interventions to encourage people with a range of MSDs [22,34] to assume a physically more active role in their management.

Pedometers have been commonly employed to provide a measurement of walking undertaken as part of a PA program, to provide patient feedback, and as a motivational instrument within intervention programs designed to increase activity and improve the quality of life, across a range of clinical conditions including: chronic obstructive pulmonary disease (COPD), [35] diabetes, [36,37] inactive overweight and obese older people, [38,39] and healthy adults [40,41]. In addition, a number of studies describe a variety of pedometer-driven walking research protocols for adults with low back pain [42,43] designed to assess the effects on pain-related disability and functional interference.

A systematic review on the effect of aerobic walking or strengthening exercises for OA of knee found walking to be effective in decreasing pain and improving function in this population [34]. Hendrick and colleagues similarly presented moderate evidence for walking interventions playing a role in decreasing pain levels in patients with acute and chronic LBP (CLBP) [22]. However, there is little standardization between protocols as to the most effective pedometer-driven walking programs for MSDs, and therefore it is difficult to evaluate the relative effectiveness of one program over another within this population. As there has been no systematic review focusing on the effectiveness of pedometer-driven walking programs as part of the management of adults with MSDs, the primary purpose of this systematic review was to investigate the evidence for pedometer-driven walking programs as an intervention in promoting PA and improving health-related outcomes when compared to no intervention, or a different type of intervention, among adults with MSDs.

### Research question

Does using a pedometer-driven walking program increase physical activity, and/or improve health in patients with MSD?

## Methods

### Search strategy

A systematic search of the literature was carried out using the following electronic databases: MEDLINE, CINAHL, Embase, Cochrane Library, PubMed, Scopus, PEDro, Web of knowledge, Sport Discus, AMED, and Science Direct. All databases were searched from their inception to 20 February 2014. Keywords and MeSH terms used in the search strategy were: “musculoskeletal diseases” OR “osteoarthritis” OR "back pain" OR "spinal pain" OR "knee pain" OR "ankle pain "OR "hip pain " OR "shoulder pain" OR "lower extremity" OR "pelvic pain" AND “walking” OR "physical activity" OR "aerobic exercise" AND “pedometers” OR “step counter” (Table 1). In addition, the reference lists of all included articles were also searched for further relevant studies that may not have been identified by the search strategy described above.

**Table 1:** Search strategy used to identify the articles

**Term category**	**Words**
Mesh terms	Musculoskeletal diseases OR “back pain" OR "spinal pain" OR "knee pain" OR "ankle pain "OR "hip pain" OR "shoulder pain" OR “osteoarthritis” OR "lower extremity” OR "pelvic pain" and “Walking”
Keyword	“Physical activity”, "aerobic exercise", “Pedometers” and “step counter”

### Study selection

The review was conducted in three steps. Firstly, the first reviewer Suliman Mansi (SM) title-screened all articles for potential inclusion. The abstracts of those studies were then independently reviewed by two reviewers (SM and Paul Hendrick, PH) and consensus sought for acceptance for review of the full-text article. In the final step, the references of all full-text articles were searched for additional articles.

### Inclusion and exclusion criteria

Studies were included if they met the following criteria: (1) randomized controlled trials (RCT) and controlled trials without randomization published in the English language, (2) restricted to adults aged 18 years and over with a MSD, (3) used pedometer-driven walking as an intervention to increase physical activity, and/or improve health outcomes (physical function, and pain), (4) studies investigating mixed disorder presentations (MSD as a primary and another disorder) were also included. Studies that investigated measurement-validity or reliability- tests of a pedometer walking program were excluded.

### Extraction of data

Data extraction for all included studies was performed by the first author (SM) and cross-checked for consensus by a second author (PH). Data related to author, year of publication, study design, objectives, sample and participants, components of pedometer intervention, mean steps per day, and outcomes were extracted and tabulated.

### Quality assessment

The methodological quality of the studies included in this review was assessed using a criteria list that has been used in previous reviews [44,45]. The criteria list (Table 2) includes four domain measurements to assess the quality of the study design (A and B); research population (C and D); quality of measurements (E, F, G, and H); and quality of analysis (I and J). These criteria were applied, with two reviewers independently evaluating the methodological quality (SM and PH). The criteria answer format included positive (+), negative (-), or unclear (?). Possible scores ranged from 0 to 10; studies that scored ≤ 5 were deemed to be low quality, and ≥ 6 represented high quality.

**Table 2:** Criteria list for the methodology quality assessments

**Item**	**Description**
**A**	**Randomization:** Is randomization described and adequately performed? Positive if a random assignment to the research groups was performed and had been described explicitly.
**B**	**Control condition:** Is there an adequate control conditions? Positive if the control group is from that same setting as the intervention group and (1) an alternative treatment was given, (2) if there was a comparable condition that controlled for a part of the intervention, (3) if usual care was given, or (4) if nothing was done.
**C**	**Research groups comparable at commencement:** Positive if the comparability of the research groups was statistically tested before the start of the intervention and the tests showed that the intervention group and control group did not differ with respect to age and at least one of the relevant outcome measures. In case the groups did differ, positive if this difference was.
**D**	**Dropout described and acceptable:** Positive if (selective) dropout was described and when dropout was <20% at short-term follow-up (6 months or less) and <30% at long-term follow-up (longer than 6 months.
**E**	**Was the person conducting the measurements blind for group assignment (or was an attempt made at baseline?):** positive if the measurements were conducted by a person blind for group assignment or if data collection was done with questionnaires that the respondent could fill out, in a situation not influenced by the researcher.
**F**	**Respondent blind for group assignment:** Positive if the respondent had (or could have had) no knowledge on the results of the group assignment.
**G**	**Timing of measurements is comparable for the different research groups:** Positive if the measurements were conducted at comparable moments for both the control group and the intervention group.
**H**	**Is the length of the follow-up described and acceptable?** Positive if a follow-up of 6 months or longer was described.
**I**	**Intention to treat-analysis:** Positive if all initially included and group-assigned participants are mentioned and analyzed in the original groups.
**J**	**Control for potential confounders:** Positive if the analysis controlled for potential confounders.

To determine the effectiveness of the interventions, a rating system comprising four levels of evidence was performed, based on a best-evidence synthesis [46] used previously for PA interventions [47,48].

• Level 1, strong evidence: multiple RCTs of high quality with consistent positive results.

• Level 2, moderate evidence: one RCT of high quality and one or more relevant low quality RCTs. Consistent positive outcomes of the studies.

• Level 3, limited evidence: only one RCT of high quality or multiple low quality RCTs. Consistent positive outcomes of the studies.

• Level 4, no evidence: only one low quality RCT, negative or contradictory outcomes of the studies, or no relevant studies.

## Results

**Figure 1:**
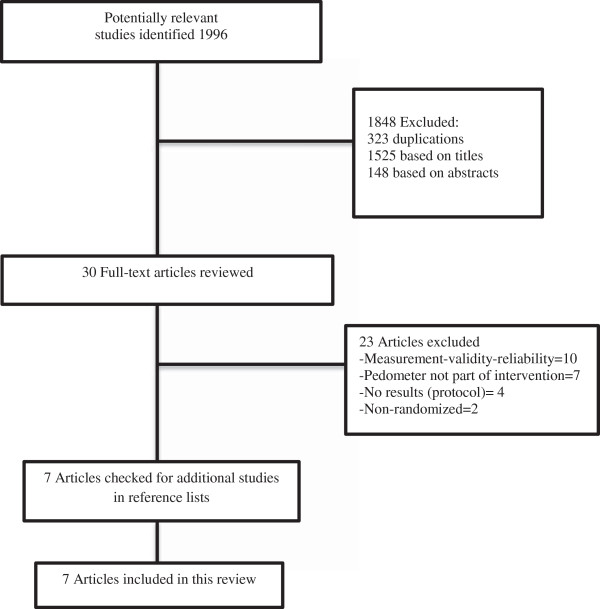
Progress through the stages of study selection.

A total of 1996 articles were retrieved using the search strategy detailed in the methods section. Based on the title, 1848 articles were excluded: 323 as duplicate titles and 1525 did not meet inclusion criteria. One hundred and forty-eight abstracts were then reviewed, leaving 30 full text articles included for review. Twenty-three of these manuscripts did not meet the inclusion criteria and were excluded due to the following reasons: Non-RCTs (2), protocol study (4), pedometer not part of intervention (7), and measurement validity/reliability (10). A total of seven articles met the full selection inclusion criteria (Figure 1). No further studies were identified from the manual search of the reference lists of included articles.

### Study characteristics

All seven included studies were of a randomized controlled trial (RCT) design and ranged in date of publication from 1998 to 2013 [49-55]. Three of the studies were conducted in the United States, [49,51,54] while two studies were conducted in Japan [52,55] one was Australian [50] and another study was conducted in Ireland [53]. The total number of participants across the seven studies was 484, with an age range of 40 to 82 years. Five of the seven studies included both males and females [49-51,53,54] while two studies [52,55] recruited females. Sample size ranged from 34 to 229, with the greatest contribution of 229 participants coming from Krein et al. [54]. The majority of included studies examined the effectiveness of a pedometer-driven walking program to increase PA levels in adults with MSDs. A summary of these studies is provided below.

Talbot and colleagues [51] compared the effectiveness of a pedometer-driven walking program with goal-setting versus an education program (no pedometer) by two registered nurses in 34 participants aged over 60 years, with osteoarthritis of the knee. Participants in both the walking intervention and control groups received a 12-hour education arthritis self-management program including 1 hour of information on exercise for coping with arthritis. Participants in the intervention group also received a pedometer, step goals, feedback, and an education booklet. Outcome measurements were evaluated at baseline, week 12 and week 24.

Ng et al. [50] compared the effectiveness of two different pedometer-driven walking program interventions called ‘Stepping Out’ on osteoarthritis symptoms of the hip and knee in 36 patients aged between 42 and 73 years, over a 24-week period. The first group (A) received a pedometer walking program three times per week plus glucosamine sulphate (GS) intake, and the second group (B) received a pedometer-driven walking program undertaken five times per week plus GS intake. Both groups received GS intake alone for 6 weeks before the Stepping Out program commenced. By the sixth week session, participants were asked to initially walk at least 1500 steps per day, and gradually increase their steps from 1500 to 3000 steps per day by the week 12 assessment. From the twelfth week, participants were asked to increase their walking to 6000 steps per day until the eighteenth week. Participants in group (B) received the same program and procedures with the same goals, but were asked to walk five days per week (Table 3). Between week 18 and the 24 week follow-up, participants were instructed to continue with either the walking program or to try another PA as an option. Results were reported at week 6, 12, 18 and the 24-week follow-up.

**Table 3:** Intervention design

**0-6 weeks**	**7-12 weeks**	**13-18 weeks**	**19-24 weeks (Follow-up)**
GS intake only	Walking up to 3000 steps per day plus GS	Walking up to 6000 steps per day plus GS	Any PA choice

GS: glucosamine supplements, PA: physical activity.

Fontaine and Haaz [49] examined the effects of a pedometer walking program on health status, pain, and PA in low-active adults with fibromyalgia syndrome (FMS). Forty-eight participants aged 18 years or older diagnosed with FMS were randomized into one of two groups: a pedometer based intervention group or a control group. The pedometer based intervention group included a 90-minute cognitive-behavioral PA program (which provided an overview of FMS, problem solving, goal setting, and self-monitoring) every two weeks for 12 weeks. At the first week, participants were asked to wear the pedometer during waking hours and to record daily step counts, while walking 10 min per day five to seven days per week, and to gradually increase daily duration of walking by five minutes every week. The control group received a 90 min fibromyalgia education program once a month over a three month period, including information on the symptoms, diagnosis, exercise and PA, and treatment for FMS, without wearing pedometers.

Toda et al. [52] examined the effects of a weight loss program for improving symptoms of OA in obese patients with knee OA. They investigated a number of variables in the weight control program (including weight, total cholesterol, triglycerides, blood glucose, serum levels of insulin, and PA by pedometer) to determine if any of these factors were associated with symptomatic relief of knee OA symptoms. Both groups received non-steroidal anti-inflammatory drugs (NSAIDs) alone for 4 weeks before the study. The intervention group then received 0.5 mg Mazindol once per day plus NSAID medication twice a day, with 150 ml low calorie soup given at both breakfast and lunch for 6 weeks; in addition this group was instructed to wear a pedometer and to walk for 30 minutes each day for 6 weeks, during both work and leisure time. The control group received the same NSAIDs as the intervention group for 6 weeks, and a pedometer walking program without any specific instructions. McDonough and colleagues [53] examined the feasibility of a pedometer walking program with education advice increasing physical activity in patients with CLBP. The study randomized 57 patients to a pedometer intervention group (n = 40) or to a control group (n = 17; education advice). All participants attended a one-hour session on education advice and completed a 10 minute self-efficacy walk at week one. Participants in the intervention group received a pedometer for 8 weeks, with individual step goals, feedback, regular contact, and education advice. Results were reported at baseline, week 9 and 6 month follow-up.

Krein et al. [54] examined the effect of a pedometer-based internet-mediated intervention on reducing CLBP at 6 and 12 months for 229 patients who were divided into an intervention group (n = 111) and a control group (n = 118). The intervention group received uploading pedometers and access to the intervention website which including goal setting, feedback, motivational messages, and social support following the Stepping Up to Health program. The control group also received pedometers but without goals or feedback. Lastly, Hiyama et al. [55] randomized 40 patients with knee osteoarthritis to a walking group including pedometer (n = 20) or a control group (n = 20) for 4 weeks. All participants attended physical therapy once a week, and completed exercises for muscle strengthening and range of motion every day at home. The walking group received instructions to increase their steps to 3000 steps more than baseline value and record their steps every day. Outcome measurements were evaluated at the week before the intervention started and at week four of the intervention (see Table 4).

**Table 4:** Studies that used pedometers as an intervention for musculoskeletal diseases

**Study**	**Objectives**	**Sample and Design**	**Pedometer intervention**	**Mean steps per day**	**Result**
				**and (pedometers used)**	
RCT	To compare the effectiveness of two walking programs in combination with GS on OA symptom and PA in patient with hip or knee OA	-36 participants (age = 42-73), randomized into two intervention groups	From 0 to 6 weeks both groups received GS.	between week 6 and 18 (3920 – 6683) steps in both groups	No differences between groups in step/day/(P = .07). Significant improvements in pain (P = .001) and physical function (P = .001) for both groups
			Group A: From 7 to12 weeks received GS + pedometer and walking up to 3000 steps/day		
		- Group A n = 19 (walking 3 days /week)			
Ng et al. [50]					
			From 13–18 weeks received GS + pedometer and walking up to 6000 steps/day Group B: same group A but walking 5 day/week	Pedometer: not mentioned	
		-Group B n = 17 (walking 5 days/week )			
		program lasted 12 weeks			
RCT	To determine whether a pedometer program with arthritis self-management would increase PA and muscle strength in subject with OA of knee.	-34 participants with knee OA (age = 60 and older)	Both groups received 12 hours arthritis self-management education (UDE) over 12 weeks	Education group ( 4652–3972)	Significant differences between groups in PA (P = .04), and muscle strength (P = .04) with no significant in pain (P = .95)
		- randomized into two groups.		Pedometer group (3519–4337)	
Talbot et al. [51]			Pedometer group received instruction to increase their step count by 10% every 4 weeks from their baseline step count with feedback and exercise materials.		
		pedometer group n = 17			
				Pedometer: New Lifesty- les Digi-walker SW-200, Yamax, Japan	
		education group n = 17			
		program lasted 12 weeks			
RCT	To compare pedometer program vs. an education program on health status and PA levels in adult with Fibromyalgia syndrome (FS).	-48 adults age (48–52 years), randomized into	Pedometer group; received 90 min cognitive behavioral program × 2 per weeks for 12 weeks. from week 1 participants were asked to increase 10- min walking every week to reach 30 min by week 5 control group: received 90 min cognitive behavioral program once a month for 12 weeks	pedometer group (2337–3970), no steps for control group	Significant increase in PA for intervention (P = .001). No significant differences between groups in pain (P = .060), fatigue (P = .85) and six-min walk (P = .92) at 6 weeks
				Pedometer: Accusplit® Eagle Activity Pedometer	
		-pedometer n = 22			
		-Control group n = 26			
Fontaine, & Haaz [49]		program lasted 6 weeks			
RCT	To determine the variable most closely related to symptomatic	-40 women mean age (63.5), randomized into intervention group n = 22 control group n = 18 The program lasted 6 weeks	Both groups received drugs (NSAID) for 4 weeks before study.	intervention (N/R -7500 steps) control (N/R-7300 steps)	No significant differences between groups in steps (P = 0.86) at 6 weeks. Correlation significant between steps and relief pain (P = .003)
			Intervention group: received 0.5 mg Mazindol once per day plus the NSAID twice a day, and instructed to wear a pedometer to walk 30 min each day for 6 weeks		
	relief of OA of knee in response to a weight				
Toda et al. [52]	control and walking program.		Control group: received same the intervention group but without any instruction or feedback on pedometer.	Pedometer: Seiko, Tokyo, Japan	
RCT	To examine the feasibility of 8 weeks pedometer with education materials on CLBP patients	57 participants (age 42-60), randomized into two groups	Both groups received a single 1 hour education session.	Intervention group: (5563–8339)	Participants in intervention increased their step count from baseline by 2776 (95% CI, 1996–3557) and improvements in pain score(ODQ) by 8.2% (CI, -13-3.4) at 6 weeks
		Pedometer group n = 40		Control group: not reported	
			Pedometer group: in week 1, 10 min self-efficacy walk completed. Week 2, meeting to provide step target, between week 3 and 8 weekly phoned to discuss the progress. This program was based on 5A^,^s framework including 1. ask/assesses barriers to PA, 2. advise to increase PA, 3. change walking goals, 4. address barriers with feedback, 5. regular feedback.		
		Control group n = 17			
				Pedometer: Yamax, Digi walker CW-701, Japan	
		program lasted 8 weeks			
McDonough et al. [53]					
RCT	To determine whether a pedometer-based internet can reduce CLBP	229 participants (age 51.9 ± 12.8), randomized into two groups	Intervention received pedometer and access to a website which provided feedback, goal setting, motivational messages and social support	Intervention(4492–5370) Control (4322–4682)	No significant differences between groups in steps at 6 and 12 months respectively (P = .12, and P = .08). Significant difference between groups in RDQ scores (P = .02) at 6 month, and non-significant at 12 months (P = .07)
		Intervention n = 111			
Krein et al. [54]		Control n = 118	Control group received pedometer without access to intervention website	Pedometer: Omron HJ-720ITC	
RCT	To examine whether a walking exercise can improves the dual-task performance in older adults with knee OA	40 participants , randomized into two groups	Both groups attended one session of physical therapy once a week, and also received ice therapy, exercises for range of motion and muscle strength at home every day.	Walking group (4453–7285)	Significant increase in PA for intervention (P = .001). Participants in intervention group significantly improved their functional disability and pain (P < 0.001, and P < 0.001 respectively)
				Control group(4425–4207)	
		Walking group n = 20 (age 71.9 ± 5.2)			
Hiyama et al. [55]					
				Pedometer:KenzLifecoder EX, Suzuken Co, Ltd, Japan	
			In addition, walking group received pedometer with instruction to increase their steps to 3000 steps more than their baseline		
		Control group n = 20 (age 73.8 ± 5.7)			
		program lasted 4 weeks			

PA: physical activity, OA: osteoarthritis, GS: glucosamine sulphate, NSAID: nonsteroidal anti-inflammatory drugs, ODQ: Oswestry Disability Questionnaire, CLBP: chronic low back pain, RDQ: Roland Morris Disability Questionnaire.

### Methodological quality assessment

Methodological quality scores are shown in the Table 5. There was 100% agreement between the two reviewers’ independent evaluation scores. The quality scores achieved ranged from 3 to 10 with five studies scored ≥ 6 out of 10 as a high quality score [50,51,53-55], and two studies scored ≤ 5 out of 10 [49,52] as low quality scores. One study achieved a maximum quality score [54], and five studies failed to score on the double blinding items (E and F); this may be due to the nature of the interventions.

**Table 5:** Methodological quality assessment scores for the included studies

**Study**	**A**	**B**	**C**	**D**	**E**	**F**	**G**	**H**	**I**	**J**	**Total**
Ng, Heesch et al. [50]	+	+	+	+	-	-	+	+	+	?	7
Talbot et al. [51]	+	+	+	+	-	?	+	-	+	-	6
McDonough et al. [53]	+	+	+	+	-	?	+	+	+	+	8
Krein et al. [54]	+	+	+	+	+	+	+	+	+	+	10
Hiyama et al. [55]	+	+	+	+	+	+	+	-	-	+	8
Fontaine and Haaz [49]	?	+	+	-	?	?	+	-	?	?	3
Toda et al. [52]	?	+	+	+	+	?	+	-	-	?	5

A: Randomization, B: Control condition, C: Research groups comparable at commencement, D: Dropout described and acceptable, E: measurements blinded, G: Respondent blinded, H: length of the follow-up, I: Intention to treat-analysis, J: Control for potential confounders.

### Pedometer intervention

From the seven pedometer walking interventions reported among patients with MSDs, four interventions focused on improving knee osteoarthritis patients [50-52,55] while two interventions [53,54] targeted CLBP patients, and one intervention [49] focused on fibromyalgia patients. The duration of the interventions ranged from 4 weeks to 12 months, with several walking protocols including the Stepping Up to Health program, [50,54] cognitive dual-tasks performance, [55] the 5As model of behavior change, [53] active living every day, [49] and the walk + program [51] as the framework for the interventions. However, the majority of those were based on Social Cognitive Theory [56] which involves using behavioral strategies such as goal setting, problem solving, self-efficacy, and social support to promote PA or to provide information on the benefits of walking to health or feedback and also to provide individual specific step goals. In all interventions, participants were instructed to use pedometer driven walking at least three times per week. The drop-out rate between baseline and post-intervention varied between 7.5% and 29.0% except one study reported no dropouts [55]. Reasons for drop-out included death, increased pain, scheduling conflicts, and forgetting to attach the pedometer for several days. All intervention studies provided information on the amount of steps per day before and after the intervention except for Toda et al. [52]. In addition, different pedometer models have been used (Digi-walker SW-200 [51]; Yamax, Digi walker DW-701 [53]; Seiko, Tokyo [52]; Omron HJ-720ITC [54]; Kenz Lifecoder [55]; and Accusplit® Eagle Activity Pedometer [49]) as a motivational tool for increasing PA and as a measurement tool across all seven studies; the details of models are shown in Table 4. However, the majority of included studies did not provide details regarding the validity and reliability of the pedometers or describe the instructions about how pedometers were used.

### Effect of interventions on physical activity levels

All studies showed positive findings for using a pedometer to increase the level of PA over the intervention periods (Table 6). Five out of seven studies showed statistically significant improvements in step count in the intervention group relative to baseline (5/7: 71%; p < 0.05). Four of these studies had high methodological quality scores [50,51,53,55] and one had a low quality score [49]. However, the majority of studies used pedometers for goal-setting to increase the daily PA level. The pedometer goal-setting regime utilized by Talbot et al. [51] resulted in significantly increased (p = 0.040) step counts from baseline (from a mean 3519 steps per day to 4337 steps per day after 12 weeks) in the pedometer group compared to control, and no decreases in activity at the 24-week follow-up. Ng et al. [50] reported an increase in PA (number of steps per day from 3920 to 6683) over the 12 weeks in both pedometer-driven walking groups between week 6 and week 18. Fontaine and Haaz [49] also found the pedometer-driven lifestyle PA group significantly increased PA levels (P = 0.001) (daily steps increasing from 2337 to 3970) at 12 weeks post-intervention in the intervention group, with no step count data available for the control group.

**Table 6:** Pedometer data at baseline and after intervention

**Study**	**Pedometer intervention**			**Control group**		
	**Baseline**	**Post-test**	**MD**	**Baseline**	**Post-test**	**MD**
	**Mean (SD)**	**Mean (SD)**		**Mean (SD)**	**Mean (SD)**	
Talbot et al. [51]	3519(2603)	4337(2903)	818	4652(2622)	3972(2563)	-680
McDonough et al. [53]	5563((N/R)	8339(N/R)	2776	(N/R)	(N/R)	(N/R)
Fontaine and Haaz [49]	2337(427)	3970(598)	1633	(N.P)	(N.P)	(N/R)
Toda et al. [52]	(N/R)	7500(N/R)	(N/R)	(N/R)	7300(N/R)	(N/R)
Hiyama et al. [55]	4453(1734)	7285(1638)	2829	4425(1627)	4207(1436)	-218
Ng et al. [50]**2 N**	3920(2441)**٭**	6683(3403)	2763	(N.C)	(N.C)	(N/R)
Krein et al. [54]	4492(2749)	5370(3180)	877	4322(2285)	4682(2925)	360

2 N = two intervention groups ٭ = for both intervention groups, N/R = not reported, N.C = no control group, N.P = pedometer not applied, SD = Standard Deviation, MD = Mean difference.

McDonough et al. [53] showed an increase in daily steps taken of 2776 on average in the intervention group over 8 weeks, but no step count data was reported for the control group. A similar increase was reported by Hiyama et al. [55], who recorded a mean difference of 2832 steps per day in the intervention group after 4 weeks of intervention, which was significantly greater than in the control group (p < 0.001). In addition, Krien et al. [54] reported that daily step counts increased by an average of 878 steps per day within the intervention group compared to an average of 361 steps per day for the control group after 6 months of intervention, but this difference was not statistically significant.

Overall, results from across six studies reported that PA, assessed by walking, significantly increased by an average of 1950 steps per day (ranging from 818 to 2829 steps) over baseline assessment. However, it should be noted that this result is simply an average of the mean increases across six studies; individual results varied markedly.

### Effect of interventions on health benefits

For secondary outcomes in this review, we found the majority of studies reported results on functional performance and pain scores. While there was a variety of measures used to assess disability and pain in these studies, the majority indicated an improvement in physical function, pain, or other health variables (fatigue, anxiety and depression) following the intervention [49-51,53-55]. Four of these studies showed a statistically significant improvement in disability and pain scores [50,51,54,55] in the intervention groups. Krein et al. [54] found that a pedometer driven walking program significantly decreased back pain-related disability as recorded by the Roland Morris Disability Questionnaire (RDQ) (p = 0.02, MD = 1.6, 95% CI, 0.3-2.8) compared with a control group at 6 months post-intervention. Similarly, Ng et al. [50] reported that the mean scores of the Western Ontario and McMaster University (WOMAC) and Osteoarthritis Index numeric rating scale (NRC) significantly improved (p = 0.001) in both groups between week 6 and the final follow-up points, with no significant difference reported between the groups in any outcome measure at any assessment point.

Hiyama et al. [55] used the Japanese Knee Osteoarthritis and Automaticity Index measures to assess participants’ functional disability and pain. The study reported significant improvements in automaticity (p < 0.001) from baseline to post-intervention for the walking group. Functional performance, muscle strength, and pain were also measured by Talbot [51] who reported a significant increase in quadriceps femoris muscle strength (p = 0.040) and functional performance (p < 0.050) in the intervention group following the intervention, with no significant difference in pain scores. McDonough et al. [53] reported a greater reduction in levels of disability (Oswestry Disability Questionnaire; ODQ) by -5.5 points (95% CI, -8.8 to -2.2) for the intervention group compared with participants assigned to the control group ( -1.0, 95% CI, -7.6 to -5.6). Toda et al. found a significant correlation between the number of steps per day and increasing symptomatic relief of knee OA pain (p = 0.003, r = -0.58) and decreasing body fat (p = 0.012, r = -0.62) [52].

## Discussion

The primary aim of this review was to identify the effectiveness of pedometer-driven walking programs in increasing levels of PA in patients with MSDs. Seven studies were identified which examined the effectiveness of pedometers to increase PA levels and improve physical function and pain in the short term. The majority of studies included in this review reported significant increases in PA (p < 0.050) following the intervention, with a mean positive change in PA of 1950 steps per day over the intervention period, and improved scores for pain and physical function after the intervention. This represents strong evidence (level 1) for the effectiveness of pedometer walking interventions for promoting PA levels in these patient populations. Evidence was appraised and synthesized based on the consistently positive results and high methodological quality of studies included in this review [44,46].

### Interventions and physical activity levels

It appears that pedometer based walking interventions with a combination of behaviour strategies are effective to increase PA behaviour among MSD populations. The majority of studies included in this review used a number of strategies to maintain the increased levels of PA within the intervention programs. These generally consisted of a range of goal setting strategies, and cognitive-behavioral approaches. For example, the study by Talbot et al. [51] reported an increase in the level of PA by 23% compared to a 16% decrease in steps in the control group. Such reported increases may be due to the targeted step goals employed, whereby participants were instructed to increase walking by 10% every 4 weeks above their baseline value. It may also be that giving feedback to patients on an individual basis and the provision of reading materials also supported the effectiveness of the intervention. These findings are consistent with previous studies targeting non-MSD conditions, including type 2 diabetes, acute coronary syndrome, and inactive populations [35,36,38,57-59] that demonstrate positive effects for a range of pedometer-driven walking interventions combined with cognitive-behavioral strategies to increase PA levels and quality of life.

Data from other studies included in this review also report similar or higher increases in PA levels after the intervention [49,50,53,55]. This finding is reflective of evidence from previous studies which suggests that combining pedometer-driven walking programs with goal setting [36,38,60] or cognitive behavioral strategies [57-59] is more effective than pedometer-driven walking alone in adult outpatients. A case study that investigated a pedometer walking intervention in patients with OA [60] showed that incorporation of goal setting into the program was effective in increasing PA levels. These findings are consistent with a recent meta-analysis of randomized controlled trials assessing promotion of PA within sedentary adults in primary care which reported the positive impact of physician counselling, written materials and advice sessions on increasing physical activity levels at 12 months [61].

The daily increase in step count between baseline and following the intervention varied across included studies. The step-count increase ranged from 818 to 2829 steps per day over the intervention, with interventions ranging from 4 weeks to 12 months. Such variability may be a reflection of the range of conditions and populations, CLBP [53,54], hip and knee OA [50-52,55] different country settings, and accuracy of the pedometers used. For future study, attention must be taken regarding the validity of pedometers for disabled populations, and in particular factors which potentially impact on the measurement of the number of steps taken during the intervention, such as pedometers that are not accurate at low speed walking [62]. Consistent with an early systematic review exploring the validity of pedometers [63] which reported that patients with disability are likely to have slower walking speeds compared to healthy controls. In addition, the reliability and validity of several pedometer models were examined in the literature [64,65] and they found the Yamax Digi-walker series pedometers (Yamax DW200 and 701, Kenz Lifecorder (KZ), New-Lifestyles NL-2000 (NL), and Omron (OM)) were the most suitable for measurement of PA in research studies; recording 1-3% error of actual steps taken at different walking speeds. Thus, choosing appropriate pedometers for these populations and determining the optimal pedometer placement is necessary to avoid methodological limitations.

The majority of studies included in this review used walking pedometer interventions in one trial arm that aimed at increasing PA levels in the short term, following a commonly applied PA recommendation: [10,66,67] i.e. involving moderate intensity activities such as walking, three to five times per week, for 30 to 60 min per session. It is significant that patients with MSDs in these studies were encouraged to meet the minimum recommended levels of PA for health related benefits [27,68]. The mean step count following the intervention ranged from 3970 to 8339 steps per day across the six studies, with an average of ≈ 5996 steps per day. The average increase of 1950 in step-counts represents more than 32% increase above baseline (range 20% to 70%), and such increments are equivalent to approximately 20 minutes walking per day. Importantly, this increase in step count was sufficient to enable participants to meet the minimum recommended levels of PA [66] of between 3500 to 5500 steps per day for people with physical disabilities [66].

Although two studies [49,53] reported significant increases in the number of steps after the intervention, they failed to report baseline and post-intervention step counts for the control group which precludes further evaluation and comparison of groups. A further study [50] also reported an increase of 2763 steps over 12 weeks of the Stepping Out program in both intervention groups, with no significant difference in the number of days per week spent walking between groups (3.07 ± 0.82 and 3.93 ± 1.09 mean days per week respectively). Thus, it appears that participation in walking either three days or four days per week were effective for increasing PA and reducing pain in this population. This result may reflect that the optimal walking period for patients with hip or knee OA is between two to four days per week in order to increase PA and improve health-related outcomes [69-71]. Equally, it may be difficult to ask such patients to walk more than three days per week, as most patients with hip or knee OA have inactive lifestyles and are three times more likely to have difficulty walking [72]. However, walking three days per week may be advantageous for people with pain and disability, allowing them time for recovery during the rest times within the week.

People living with disability such as MSDs are usually classified as a low activity population [73], which may be deleterious to their health and affect their ability to walk if the sedentary behavior is sustained [73]. In this review we observed that all studies reported a low number of steps at baseline ranging from 2337 to 5563 steps per day, except one study with no assessment at baseline reported [52]. This mean steps/day places these participants within the low active category (<5000 steps per day) [74]. The increase in step count following the pedometer driven intervention in these studies is a reflection of an inactive population significantly improving their PA [75] and potentially indicates that a cognitive-behavioral PA program may be a key motivational factor for increasing PA behavior. These findings suggest that pedometer based interventions in combination with behavioural strategies are effective in PA behaviour change (short-term) to motivate and track progress of their steps. This finding is consistent with a systematic review which found evidence to support the efficacy of behavioural interventions for promoting PA to sedentary adults in primary care [61].

### Interventions and health benefits

Previous research has shown that physical activity interventions improve overall health, with overall reduced general risk of premature mortality, [10] decreasing pain, and improving function with disability [34] across all groups and both sexes in the general population. Based on our data, significant improvements in disability and pain scores [50,51,54,55] were reported in the intervention groups. Talbot et al. [51] reported a 21% increase in quadriceps femoris muscle strength in the intervention group compared to a 3.5% decrease in the control group. This result may in part be due to an increased step count in the intervention group and also potentially as a result of the 10% reduction in pain; however, there were no significant differences between the two groups for pain and physical function.

One study [52] reported that the number of steps per day was significantly correlated with symptomatic relief of knee OA pain and body fat loss after the intervention. This result may be due to the observed relationship between reductions in obesity levels and improved pain of knee OA [76,77] and is also consistent with a meta-analysis of pedometer-based walking interventions that reported a weight loss of 1.27 kg (95% CI, 1.85 to 0.70 kg) through increasing PA in the short term, with more weight loss correlated with longer programs [78]. Furthermore, a number of previous studies have shown a clear link between increased PA and weight reduction, with symptomatic relief of knee OA pain [79-81].

Step count improvements were also correlated with a reduction in back pain related disability, symptoms of anxiety and depression, fatigue, and six-min walk distance in the short term [49,53,54]. These results are consistent with many similar walking intervention studies [19,34,60,68,82,83] that demonstrated a positive effect of increased PA on pain and health-related outcomes in subjects with MSDs. Future studies are required to establish these findings within large powered clinical trials.

This review found strong evidence (level 1) for the effectiveness of pedometer walking interventions in increasing PA levels for patients with MSDs. Future research of high methodological quality with larger sample sizes is needed to investigate the effectiveness of pedometer-driven walking for improving PA and health-related outcomes in patients with MSDs. Such research is already underway: recently, two protocols have been published for controlled studies on patients with LBP using pedometer-driven walking programs [42,43] in order to reduce pain-related disability and functional limitation.

### Study limitations

This study has a number of limitations: variation in methodology, outcome measures, statistical methods and study subjects in the reviewed studies preclude a formal meta-analysis of the available data. We used four key elements of the design of the included studies to assess heterogeneity: the patients, interventions, outcomes and methods. Therefore, a narrative systematic review was conducted displaying information retrieved from the seven studies included in this review.

We also restricted our search to full–text articles in the English language, as well as the use of a limited keyword search and excluded doctoral theses and conference abstracts. This may have resulted in some relevant studies being missed. In addition, we calculated the mean number of steps as simply an average of the mean increase across six studies. Interpretation of the data and generalization of the findings should be considered; individual results varied markedly. Based on the data, the majority of studies included in this review also provided education programs such as: information on the benefits of PA, arthritis self-management program, LBP back book, and receiving medication to relieve their pain. These components may also have changed (increased) their physical activity habits leading to improvements in health outcomes.

The studies that we identified used a variety of different pedometer brands; however, the majority did not report sufficient information on the validity and reliability of pedometers used. The majority of studies included in this review mentioned the name of pedometers without providing any details on the validity and reliability of pedometer used. Several studies have been conducted to examine the validity and reliability of various pedometers under a variety of conditions to define the most accurate brand in adult populations. A study of adults [64] that compared thirteen models of pedometers under free-living conditions reported that the validity and reliability differed. We believe that the lack of information on the psychometric properties of the pedometers may have affected the report of step count levels, and hence the interpretation of physical activity.

### Further research direction

Future research should explore the long-term effectiveness of pedometers in increasing PA levels in these populations. The majority of the studies included in our review included short term follow up, highlighting the necessity of longer term studies. Pedometer-driven walking interventions also need to be compared as single interventions, and ideally against current best treatments for patients with MSDs within an RCT design.

## Conclusions

This review showed that the majority of studies reported a positive change in PA, providing strong evidence for the effectiveness of pedometer walking interventions in increasing PA levels for patients with MSDs. Improvements in physical function and pain scores were also noted in these study populations. It would appear that a combination of interventions using the pedometer, underpinned by cognitive-behavioral approaches to behavior change, are likely to be more effective in increasing PA than the use of a singular intervention approach, which is consistent with recommendations from previous systematic reviews. Further research is required to support the role of pedometer walking interventions as a long term intervention for management of MSDs.

## Competing interests

The authors declare that they have no competing interests.

## Authors’ contributions

SM and SM performed the literature search and all authors participated in the planning and design of the study and developed a search strategy. All authors read and corrected draft versions of the manuscript and approved the final manuscript.

## Pre-publication history

The pre-publication history for this paper can be accessed here:

http://www.biomedcentral.com/1471-2474/15/231/prepub
